# Case Report: Plastic bronchitis temporally associated with bordetella pertussis infection in a vaccinated child

**DOI:** 10.3389/fmed.2026.1848125

**Published:** 2026-06-24

**Authors:** Haiting Lin, Mingfei Zhong, Yuan Tu, Yueyuan Li

**Affiliations:** Department of Pediatrics, The People's Hospital of Cenxi City, Wuzhou, Guangxi, China

**Keywords:** atelectasis, bordetella pertussis, bronchoscopy, case report, plastic bronchitis

## Abstract

**Objective:**

To describe the clinical presentation, diagnostic process, management, and potential mechanisms of plastic bronchitis (PB) temporally associated with Bordetella pertussis infection in a vaccinated child.

**Methods:**

This case report follows the CARE (Case Report) guidelines. We describe the clinical course of a 6-year-old boy with PB complicating lower respiratory tract infection, including clinical features, laboratory findings, imaging, bronchoscopy, bronchoalveolar lavage fluid (BALF) pathogen detection, treatment, and follow-up.

**Results:**

The patient presented with a 4-day history of cough and fever without the classical inspiratory “whoop” of pertussis. He had completed routine pertussis immunization. Chest computed tomography (CT) demonstrated right middle lobe atelectasis with bilateral pulmonary inflammation. Despite symptomatic improvement following empirical antimicrobial therapy, atelectasis persisted. Flexible bronchoscopy revealed obstructive mucopurulent airway casts consistent with PB, which were removed. BALF pathogen detection identified Bordetella pertussis and Rhinovirus A as high-confidence pathogens, with Aspergillus fumigatus and Epstein–Barr virus detected at lower confidence levels. After bronchoscopic intervention and pathogen-directed therapy, symptoms resolved completely and imaging confirmed lung re-expansion. No recurrence was observed during 6 months of follow-up.

**Conclusion:**

This case reports a potential temporal association between PB and Bordetella pertussis infection in a vaccinated child with atypical clinical features. To our knowledge, this is the first documented case of PB temporally associated with B. pertussis. However, causality cannot be definitively established due to the presence of copathogens and the low sequence count of the bacterium. Early bronchoscopy with BALF analysis should be considered in children with pneumonia complicated by persistent atelectasis or poor response to empirical therapy.

## 1 Introduction

Plastic bronchitis (PB), also known as cast bronchitis, is a rare but potentially life-threatening respiratory condition characterized by the formation of branching intrabronchial casts that obstruct the airways (1). These casts are typically composed of mucus, fibrin, and inflammatory cellular debris. PB can lead to significant ventilation impairment and, if not promptly recognized, may progress to respiratory failure (2).In pediatric populations, PB most commonly occurs in association with congenital heart disease following surgical correction, immune-mediated or inflammatory disorders, and severe respiratory infections (3). Among infectious causes, adenovirus (particularly types 7 and 11), Mycoplasma pneumoniae, and influenza viruses have been most frequently reported. These pathogens are thought to induce intense airway inflammation, epithelial injury, and mucus hypersecretion, thereby promoting cast formation. Nevertheless, the full spectrum of infectious agents associated with PB remains incompletely defined.

Bordetella pertussis, a Gram-negative coccobacillus, is the causative agent of pertussis (whooping cough), a highly contagious respiratory illness (4). Classical pertussis follows a triphasic course comprising catarrhal, paroxysmal, and convalescent stages, with severe paroxysmal coughing and a characteristic inspiratory “whoop” during the paroxysmal phase. However, in vaccinated individuals, pertussis often presents atypically, with attenuated or nonspecific symptoms, which may delay diagnosis and complicate clinical recognition.

To date, PB associated with Bordetella species has been reported only rarely. A single case of PB linked to Bordetella parapertussis has been described, and no prior reports have clearly documented PB associated with Bordetella pertussis. Here, we report a pediatric case of PB temporally associated with B. pertussis infection in a vaccinated child. To the best of our knowledge, this is the first documented case of PB temporally associated with Bordetella pertussis infection. This report adheres to CARE guidelines and aims to highlight diagnostic challenges, management considerations, and potential mechanisms underlying infection-associated PB.

## 2 Case report

A 6-years-old boy was admitted in May 2025 with a 4-day history of cough and fever. The cough was paroxysmal and difficult to expectorate but was not accompanied by an inspiratory “whoop.” Associated symptoms included nasal congestion and rhinorrhoea. The maximum recorded body temperature was 39.5 °C. There was no dyspnoea, post-tussive vomiting, chest pain, or haemoptysis. The child had no significant past medical history and had completed routine childhood immunisations, including pertussis vaccination. Oral second-generation cephalosporins had been administered to the patient at a local clinic prior to admission. Initial serological testing showed markedly elevated IgM against Mycoplasma pneumoniae (>300 AU/ml).

On examination, the patient appeared lethargic. Vital signs were: temperature 38.7 °C, heart rate 122 beats/min, respiratory rate 36 breaths/min, and body weight 20 kg. Oropharyngeal congestion was present. Chest auscultation revealed coarse breath sounds bilaterally, reduced breath sounds over the right lung, and scattered moist rales. Cardiovascular and abdominal examinations were unremarkable.

Laboratory investigations demonstrated a white blood cell count of 13.54 × 10^9^/L (neutrophils 4.09 × 10^9^/L, lymphocytes 8.02 × 10^9^/L), C-reactive protein 4.2 mg/L, and procalcitonin 0.165 ng/ml. Hemoglobin and platelet counts were within normal limits. Liver and renal function tests were largely normal, apart from a mildly elevated alanine aminotransferase level (98.6 U/L) and low serum creatinine (29.1 μmol/L). Immunoglobulin levels, complement components, anti-streptolysin O titer, rheumatoid factor, and coagulation parameters were within reference ranges.

Pathogen screening of nasopharyngeal swabs for influenza A and B viruses, adenovirus, respiratory syncytial virus, and parainfluenza viruses (types 1 and 3) was negative. Sputum polymerase chain reaction (PCR) testing for Mycoplasma pneumoniae and Mycobacterium tuberculosis was negative, and sputum culture for Haemophilus species yielded no significant growth. Serological testing for Epstein–Barr virus (EBV) and tuberculosis antibodies was negative.

**Figure 1:**
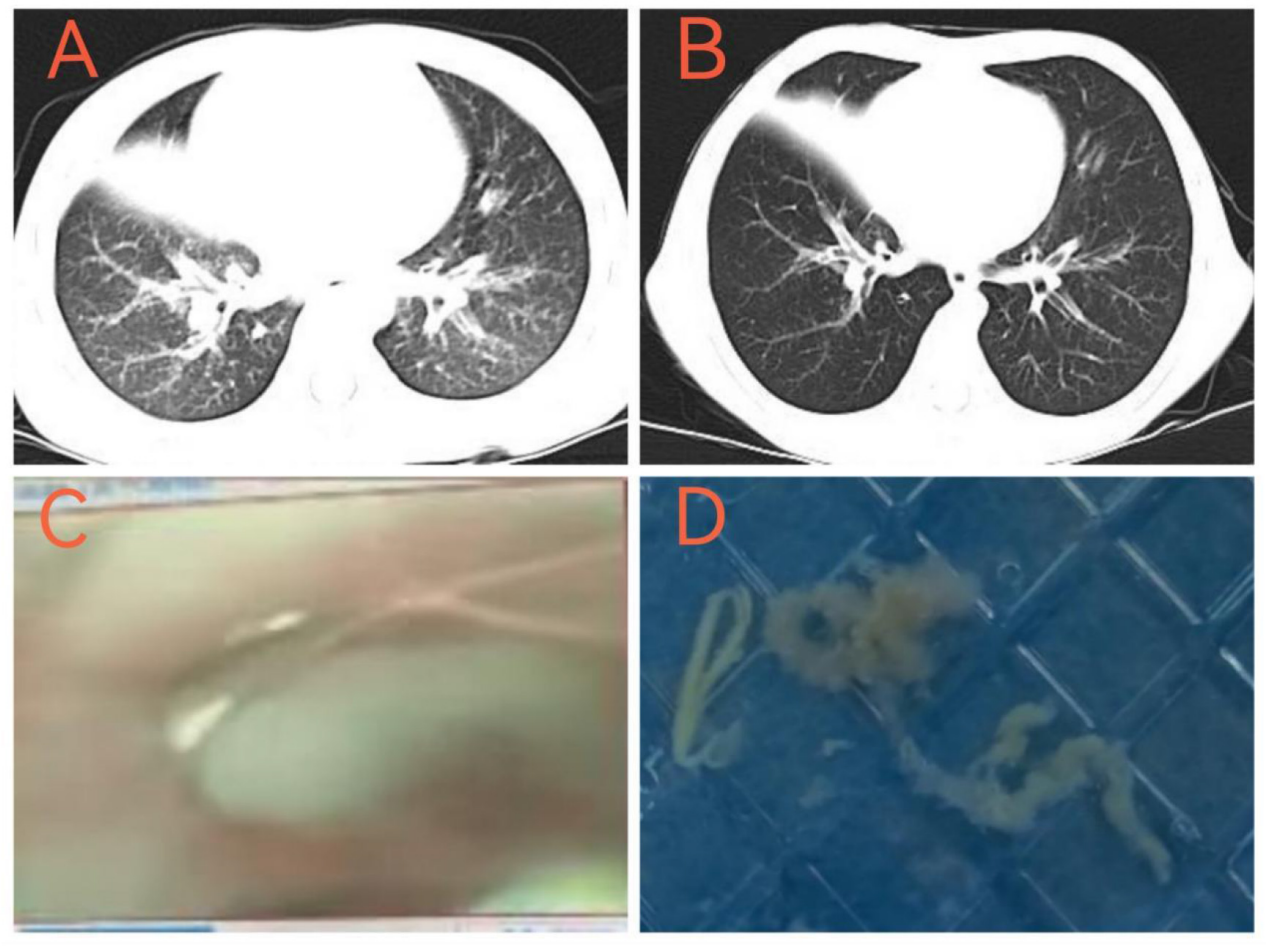
**(A)**, Chest CT scan on the day after admission, revealing atelectasis of the right middle lobe; **(B)**, Follow-up chest CT scan on day 9 of hospitalization, showing persistent atelectasis of the right middle lobe; **(C)**, Bronchoscopic image demonstrating the right middle lobe bronchus obstructed by white gelatinous material; **(D)**, Image showing the cast embolus extracted from the right middle lobe bronchus.

Chest CT performed on the day after admission revealed right middle lobe atelectasis with bilateral inflammatory changes (Figure 1). Flexible bronchoscopy was initially recommended but declined by the family. Empirical treatment with piperacillin–tazobactam and azithromycin was initiated, together with intravenous methylprednisolone, inhaled corticosteroids, and adjunctive traditional Chinese medicine. Fever and cough improved; however, diminished breath sounds over the right lung persisted.

A repeat chest CT on day 9 of hospitalization demonstrated partial resolution of pulmonary inflammation, but persistent right middle lobe atelectasis with reduced lung volume (Figure 1). After further discussion with the parents, consent was obtained for bronchoscopy. On day 11 of hospitalization, flexible fibreoptic bronchoscopy with bronchoalveolar lavage was performed under local anesthesia. Yellowish-white mucopurulent casts obstructing the right middle lobe bronchus were visualized and completely removed, consistent with PB (Figure 1). Breath sounds over the right lung improved immediately after the procedure.

BALF pathogen detection using targeted next-generation sequencing (tNGS) identified Bordetella pertussis and Rhinovirus A as high-confidence pathogens, while Aspergillus fumigatus and EBV were detected at lower confidence levels (Table 1). Following bronchoscopic intervention and adjustment of therapy based on these findings, respiratory symptoms resolved completely. Follow-up chest radiography confirmed full re-expansion of the right middle lobe. The patient was discharged on day 14 of hospitalization, and no recurrence or respiratory abnormalities were observed during 6 months of follow-up (A clinical timeline according to the CARE guidelines is presented in Table 2).

**Table 1:** Pathogen Targeted Next-Generation Sequencing (tNGS) results of Bronchoalveolar lavage fluid.

Microorganism type	Genus name	Microorganism name	Normalized sequence count	Microorganism detection concentration (copies/mL)	Pathogenicity classification
1. Special Pathogen List (Mycobacteria, Mycoplasma, Ureaplasma, etc.)					
Not detected					
2. Bacterial list					
Gram-negative Bacteria	Bordetella	Bordetella pertussis	24	< 10 × 103	A
3. Fungal list					
Fungi	Aspergillus	Aspergillus fumigatus	944	1.2 × 10^4^	B
4. Viral list					
RNA Virus	Enterovirus	Rhinovirus A	22,843	>10 × 10^6^	A
DNA Virus	Lymphocryptovirus	Epstein-Barr virus	4,094	3.5 × 103	C

Explanation of Pathogenicity Classification.

Class A: Obligate or clinically prevalent pathogenic microorganisms in respiratory specimens.

Class B: Opportunistic pathogenic microorganisms in respiratory specimens. Infections are prone to develop in patients with systemic/local immune dysfunction, impaired respiratory barrier function, or dysregulated lower respiratory tract microecology. The pathogenicity should be comprehensively judged in combination with the patient's clinical manifestations and conditions.

Class C: Normal respiratory flora that generally do not induce infection, but may cause lung abscess via aspiration.

**Table 2:** Clinical timeline (according to CARE guidelines).

Time point	Event
Day 1	Onset of cough and fever
Day 4	Hospital admission; first chest CT performed
Day 4–9	Empirical therapy (piperacillin-tazobactam + azithromycin + corticosteroids)
Day 9	Repeat chest CT showing persistent atelectasis
Day 11	Flexible bronchoscopy with cast removal; BALF sent for tNGS
Day 12	tNGS results available (B. pertussis, Rhinovirus A, A. fumigatus, EBV)
Day 14	Discharge
6 months post–discharge	Follow–up: no recurrence

### 2.1 Targeted next-generation sequencing (tNGS) of bronchoalveolar lavage fluid

#### 2.1.1 Principle of detection

The tNGS assay (Guangxi Kingmed Center for Clinical Laboratory) used in this case is a multiplex PCR-based amplification and high-throughput sequencing method for pathogen nucleic acid detection. The technical workflow includes: targeted extraction of pathogen nucleic acids using multiplex specific primers, amplification and enrichment via multiplex PCR, high-throughput sequencing of the amplified products, and bioinformatic alignment against a reference microbial database for pathogen identification.

#### 2.1.2 Reporting thresholds and strategies

Currently, there is no unified industry standard or expert consensus on positive thresholds for NGS-based pathogen detection, primarily because different pathogens vary significantly in nucleic acid extraction efficiency, genome size, and clinical pathogenicity. To assist clinical interpretation, the laboratory classifies common respiratory pathogens into three categories based on pathogenicity:

Class A (highly pathogenic): e.g., Bordetella pertussis, Mycobacterium tuberculosis, influenza virus, Legionella. These pathogens have strong clinical pathogenicity and are reported even at low sequence counts, following a sensitivity-prioritizing approach to avoid missing high-risk pathogens.

Class B (opportunistic pathogens): e.g., Aspergillus, Candida. Interpretation requires correlation with the patient's immune status and clinical presentation.

Class C (normal commensals): e.g., Epstein-Barr virus. Higher sequence counts are required for reporting to avoid overinterpretation of colonizing organisms.

#### 2.1.3 Interpretation of normalized sequence counts

According to the testing laboratory's official guidelines, the normalized sequence count reflects the strength of the detection signal—i.e., the confidence that the microorganism's nucleic acid is present in the sample. A higher normalized sequence count indicates higher detection confidence, but it does not correlate with pathogenicity. The sequence count cannot distinguish between nucleic acid from live versus dead microorganisms, nor can it definitively determine whether a microorganism is directly causing infection. When assessing whether a detected microorganism is pathogenic, priority should be given to the intrinsic pathogenicity of the organism itself. For highly pathogenic organisms (Class A), even a low sequence count should be considered clinically relevant. For opportunistic pathogens (Class B) or normal commensals (Class C), even higher sequence counts require correlation with the patient's immune status and clinical presentation.

#### 2.1.4 Detection results in this case

Bordetella pertussis is a Class A highly pathogenic pathogen, and the assay targets its IS481 insertion sequence. The normalized sequence count was 24 (positivity threshold ≥1 per 100,000 raw reads), with a detection concentration of < 1.0 × 103 copies/ml.

#### 2.1.5 Literature review:

PB associated with Bordetella infection is exceedingly rare. A single reported case described PB associated with Bordetella parapertussis in a 4-year-old girl with concomitant EBV infection (5). That patient presented with fever, paroxysmal cough, and subconjunctival hemorrhage, with right middle lobe involvement on imaging. Bronchoscopic removal of airway casts plus antimicrobial therapy with ceftriaxone and azithromycin achieved a complete recovery in the patient. To our knowledge, no previous cases have documented PB temporally associated with Bordetella pertussis infection.

## 3 Discussion

To our knowledge, this is the first reported case of plastic bronchitis temporally associated with Bordetella pertussis infection. The only previously reported case of PB associated with a Bordetella species involved Bordetella parapertussis in a 4-year-old girl (5). The present case therefore brosdens the spectrum of Bordetella-associated PB to include B. pertussis, with the important caveat that causality cannot be definitively established due to the presence of copathogens (particularly Rhinovirus A) and prior antibiotic exposure.

PB is characterized by the formation of obstructive airway casts composed of mucus, fibrin, and inflammatory cellular debris (1). In children without underlying cardiac disease, infection-related PB is increasingly recognized, although the mechanisms underlying cast formation remain poorly understood. In infection-associated PB, cast formation appears to involve eosinophil activation and extracellular trap release, particularly in influenza-associated cases. In mycoplasma pneumoniae-associated PB, neutrophil-mediated inflammation and dysregulated immune responses are thought to contribute to cast formation (6).

In this case, multiple pathogens were detected in BALF. Bordetella pertussis, a Class A highly pathogenic pathogen, was detected at a normalized sequence count of 24 (positivity threshold ≥1). Importantly, as noted in the Methods section, normalized sequence counts reflect detection confidence rather than pathogenicity, and priority should be given to the intrinsic pathogenicity of the organism itself when interpreting tNGS results. Several factors explain the low sequence count of B. pertussis. First, B. pertussis is a fastidious Gram-negative coccobacillus, and its nucleic acid extraction efficiency is inherently lower than that of many other respiratory pathogens (7). Second, and most importantly, the patient had received azithromycin prior to BAL sampling; azithromycin is active against B. pertussis and would have significantly reduced the bacterial load before the sample was obtained. Thus, the low sequence count most likely reflects the organism's fastidious nature and prior antibiotic exposure rather than indicating a low-abundance infection with limited clinical relevance. Therefore, despite its low sequence count, B. pertussis should be regarded as a likely causative pathogen in this case.

Rhinovirus A was detected at a much higher normalized sequence count (22,843 reads, Class A). However, it is important to note that normalized sequence counts in tNGS are semi-quantitative and cannot be directly compared across different pathogen species. Variations in primer amplification efficiency, amplicon length, and target gene copy number, as well as inherent differences between RNA and DNA detection, can substantially affect observed sequence counts independent of true infection burden. Rhinovirus A is a known cause of lower respiratory tract infection in children and can induce airway epithelial injury, mucus hypersecretion, and inflammatory cell recruitment (8), but it typically causes self-limited illness and has not been established as a primary cause of PB. Accordingly, while Rhinovirus A may have contributed to airway epithelial injury, it is unlikely to be the sole or primary cause of PB in this case.

Therefore, while acknowledging the limitations of a single case with copathogens, the preponderance of evidence—including the intrinsic high pathogenicity of B. pertussis, the temporal sequence of events, and the patient's clinical response to targeted therapy—supports B. pertussis as the most likely contributor to PB pathogenesis in this patient. Rhinovirus A may have played a contributory or synergistic role by creating a permissive airway environment. The detection of Aspergillus fumigatus and EBV at lower confidence levels most likely represents airway colonization or incidental viral reactivation rather than primary pathogenic involvement (9).

We hypothesize that several factors may have contributed to PB development in this patient. First, infection-related airway epithelial injury and impaired mucociliary clearance may have promoted mucus retention. Second, attenuated or atypical cough in vaccinated children with pertussis-like illness may reduce effective clearance of airway secretions, favoring cast formation. Third, accumulation and organization of mucus, fibrin, and inflammatory cells may result in the formation of bronchial casts. These mechanisms remain speculative and underscore current gaps in understanding the pathogenesis of infection-associated PB.

The markedly elevated M. pneumoniae IgM (>300 AU/ml) in the setting of negative sputum PCR and negative tNGS for M. pneumoniae warrants comment. False-positive IgM can occur due to cross-reactivity with other pathogens or persistent antibodies from prior infection. Empirical azithromycin was initiated partly to cover atypical pathogens, including M. pneumoniae, given the serological finding. It is possible that azithromycin suppressed M. pneumoniae below the detection limit of tNGS by the time BALF was obtained. However, the absence of M. pneumoniae on tNGS—despite its high sensitivity—argues against it being a dominant pathogen in this case.

### 3.1 Limitations

Several limitations should be acknowledged. First, histopathological examination of the airway cast was not performed. Histologic subtyping of PB (cellular vs. fibrinous) has prognostic and therapeutic implications, and its absence limits our ability to fully characterize this case. Second, the diagnosis of pertussis was based solely on tNGS of BALF without confirmatory nasopharyngeal PCR or paired serology (pertussis toxin IgG). Third, the low sequence count of B. pertussis and the presence of copathogens (particularly Rhinovirus A) preclude a definitive causal attribution. Fourth, the generalizability of findings from a single case is inherently limited.

## 4 Conclusion

This case represents, to our knowledge, the first report of plastic bronchitis temporally associated with Bordetella pertussis infection. The case reports a potential temporal association between PB and B. pertussis infection in a vaccinated child, though causality cannot be definitively established. This case also highlights the limitations of empirical anti-microbial therapy alone in resolving airway obstruction. Although systemic symptoms improved with antibiotics, atelectasis persisted until bronchoscopic removal of casts, suggesting that persistent lobar collapse may warrant consideration of early airway evaluation to assess for mechanical obstruction in selected patients.

## Data Availability

The datasets presented in this study can be found in online repositories. The names of the repository/repositories and accession number(s) can be found in the article/supplementary material .
